# Team management in critical care units for patients with COVID-19: an experience from Hunan Province, China

**DOI:** 10.1186/s13054-020-02921-7

**Published:** 2020-06-06

**Authors:** Li Tang, Xian-Mei Zhao, Xiao-Yan Yu

**Affiliations:** 1 Intensive Care Department, the Second Xiangya Hospital, Central South University, Changsha, Hunan Province China; 2 Xiangya Nursing School, Central South University, Changsha, Hunan Province China; 3 Clinical Nursing Teaching and Research Section, the Second Xiangya Hospital, Central South University, Changsha, Hunan Province China

The COVID-19 caused an outbreak of respiratory disease and was first found in Wuhan, China, in December 2019 [1]. To cope with this emergency, designated hospitals for COVID-19 patients were set up quickly in China, including Hunan Province which is adjacent to Hubei Province. Those severely ill COVID-19 patients require centralized care in the constructed temporary critical care unit of the designated hospital. Intensivists, nurses, respiratory therapists, infection control experts, and administrative staff form the main core of the special intensive care team. As the critical care team members have multidisciplinary background, it is a big challenge to organize the nursing process. We therefore consulted local critical care and infection control experts in environment-based risk assessment and proposed an integrated and adoptable approach.

## Clarify human resource structures

**Fig. 1:**
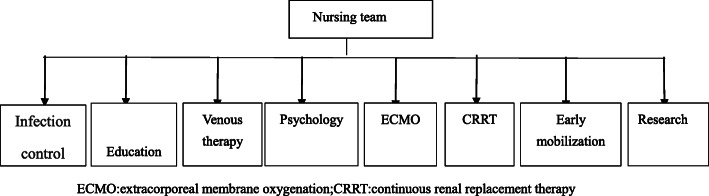
The distribution of nursing team

We built a personnel file including learning background, working years, technical title, original department, speciality, and experience of critical care. We formulated a shift responsibility for the “three areas” namely contaminated area, semi-polluted area, and clean area. According to the competence of the preliminarily assessed nurses, we hierarchically divided the frontline nurses working in the contaminated area into groups. Each group adopted the system of group leader responsibility. Hypoxemia is the main clinical symptom in patients with severe COVID-19 pneumonia, and in worse conditions, we see complications such as acute respiratory distress syndrome, septic shock, and metabolic acidosis. Bleeding and coagulation dysfunction may also occur [2]. In order to nurse the patient precisely, we set up special nursing team as follows (Fig. 1).

## Standardize communication procedures

Nurses make wireless contact with physicians and non-physician in different areas. To minimize the risk of infection, the shift time of a frontline nurse is 4 h. Due to the high frequency of handover and different working styles, the nurses confronted communication hazards. It is therefore necessary to establish a standard mode to ensure that efficient communication.

The SBAR (situation-background-assessment-recommendation) communication mode has been widely used in different areas [3, 4]. We constructed the standard mode in shift exchange, nurse-nurse, and nurse-physician communication scenarios (see Supplemental file 1). Before its initiation, a 45-min didactic training session including a PowerPoint presentation designed to address detailed principles on SBAR and a scenario simulation training was carried out to augment trainee ability.

## Secure healthcare workers

In this anti-virus war, a priority is to protect the healthcare personnel from exposure [5, 6]. Nurses are at high risk of exposure to the virus as they carry out the necessary intensive nursing. The following measures were adopted to protect nurses from infection.

### Using 6S methodology guided with the control infection principle

6S stands for Seiri, Seiton, Seiso, Seiketsu, Shitsuke, and Security. We combined this concept with the control infection principle to form the organizational safety culture which is the important level of the hierarchy in preventing cross-infection. We classified the necessity of items in different areas into three levels. The evaluation index includes the function and the frequency of use. If unnecessary, we clean it up. The residual items were set up in prescribed position with clear identification. The environment was cleaned and disinfected without any blind spots, especially in the contaminated area. All staff had to undergo infection control training before practicing clinically. We also developed a series of cleaning and disinfecting procedures structured in eye-catching easy positions and arranged infection control personnel to supervise and guide the quality of implementation.

### Restriction management of high risk procedures

Donning and doffing of personal protective equipment are a common and particularly important procedure. We put the procedure illustration on the wall, and a personnel monitored the nurses in the buffer area. Some nursing operation may generate aerosol, for example, sucking sputum, acquiring a sputum sample, oral care, nebulizing treatment, assisting with bronchoscopy, and weaning the ventilator. Current recommendations are to follow contact and droplet precautions and airborne precautions when performing aerosol-generating procedures [7]. The checklists and education program were used before the nurse applied these procedures. We also emphasized the importance of management and the evaluation of analgesia and sedatives to comfort the patient.

### Flexible shifts

Nurses who have worked in contaminated areas need to wear cumbersome PPE. Most of the patients who required intensive care support were elders with multiple comorbidities including cardiovascular, cerebrovascular, endocrine, digestive, and respiratory diseases [8]. Heavy load and long shift could increase the risk of infection. The shift time of frontline nurse was curtailed to 4 h, The second-line nurse should do continuous work for 6 h, and the shifts of nurses working in clean areas need to be changed every 12 h. The nurse should monitor the healthy situation every day and report any symptom such as fever, chills, myalgia, sore throat, runny nose, cough, vomiting, diarrhea, or pneumonia.

## Satisfy healthcare workers

Up to now, the government has issued a series of policies and communities have provided social support to care for the medical staff and their relatives. Nurses faced multipsychological stress from working in a strange environment, coping with a new infectious disease, and getting away from their family. The balance of the basic psychological satisfaction is easily broken and can lead to negative outcomes. We adopted measures based on autonomy, competence, and relatedness support. We chose an “open door” policy to give nurses the opportunity to speak out in the case of a hazard and encouraged them to seek social support from supervisors and co-workers within the organization. We allotted tasks according to their competence and willingness. The education program was formulated in terms of clinical and personal requirements to enhance capability. To relieve anxiety and fatigue, if possible, we encouraged nurses to combine their interests with the nursing work.

In conclusion, COVID-19 has caused epidemic and carrying substantial morbidity and mortality. The strategy under discussion was designed to gather a provisional and effective nursing team in a short time. We hope that our preliminary experience can improve nursing team management globally in coping with the potential infectious disease outbreak.

## Supplementary information

**Additional file 1.**

## Data Availability

Supplement file 1.
