# Supplementary material for: Team management in critical care units for patients with COVID-19: an experience from Hunan Province, China
Source: Crit Care. 2020 Jun 6;24:304. doi: 10.1186/s13054-020-02921-7 (PMC7275843; doi:10.1186/s13054-020-02921-7)
Supplement: Supplementary file 1 — Additional file 1. [file 13054_2020_2921_MOESM1_ESM.docx]

| **SBRA communication（Nurse-Physician）**  Call time respondent time | | |
| --- | --- | --- |
| **S (Situation)** | The calling is for （Bed number and Patient name） | |
|  | I am the call physician for the problem  （the reason that you are calling the physician） | |
| **B**  **(background)** | The situation began at （time） | |
|  | A brief relevant story for previous condition (Details such as any therapy received, vital signs) | |
| **A**  **(assessment)** | Patient vital signs (RR, respiratory form, HR, heart rhythm, pulse rate, BP, SPO2, temperature) | |
|  | If need to report | Patient complaint Psychological state |
|  |  | Pupil and Glasgow coma scale |
|  |  | Analgesia and sedation level |
|  |  | The intake （ml） and output （ml） |
|  |  | The treatment situation and history |
|  |  | The skin color |
|  |  | The drainage tube （volume, colour） |
| **R**  **(recommendation)** | I would like you to （content and time） | |
|  | What do you want me to do (content and time) | |
|  | The target that you want to achieve is （content and time） | |
| **SBRA communication （Handover shift）**  Bed number Patient name ID | | |
| **S (Situation)** | Diagnosis is | |
|  | Current therapy  (such as Oxygen, IV medicine, Oral medicine, Nutrition support, Nebulizing treatment) | |
| **B**  **(background)** | Admission time | |
|  | A brief and relevant summary of the patient’s story (second diagnosis such as hypertension, diabetes) | |
|  | A brief and relevant summary of the patient’s previous condition (such as any therapy received or their vital signs) | |
| **A**  **(assessment)** | Nucleic acid detection results and times | |
|  | CT results | |
|  | Patient vital signs （HR, RR, BP, SPO2, temperature, conscious/unconscious） | |
|  | Analgesia and sedation level_________________________________ | |
|  | Patient complaint Psychological situation | |
|  | Pupil and Glasgow coma scale | |
|  | The intake （ml） and output （ml） | |
|  | The skin situation  The drainage tube | |
| **R**  **(recommendation)** | Priority details for monitoring (such as vital signs, treatments, psychology, complication prevention) | |
|  | The nursing measures (Such as body positive changes, airway management, early mobilization) | |

| **SBRA communication（Nurse-Nurse）**  Call time respondent time | |
| --- | --- |
| **S (Situation)** | The calling is from |
|  | I am calling you for the problem  （reasons such as preparing materials, medicine） |
| **B**  **(background)** | A brief summary of the relevant information (eg: what is the material or drug used for ) |
| **A**  **(assessment)** | A risk assessment  (eg: the time for which the remaining material or drug may last) |
| **R**  **(recommendation)** | I would like you  （content and time） |
